# Cystatin C influences the autoimmune but not inflammatory response to cartilage type II collagen leading to chronic arthritis development

**DOI:** 10.1186/ar3298

**Published:** 2011-03-28

**Authors:** Alexandra Bäcklund, Meirav Holmdahl, Ragnar Mattsson, Katarina Håkansson, Veronica Lindström, Kutty Selva Nandakumar, Anders Grubb, Rikard Holmdahl

**Affiliations:** 1 Division of Medical Inflammation Research, Department of Medical Biochemistry and Biophysics, Karolinska Institute, Scheeles väg 2, 171 77, Stockholm, Sweden; 2 Division for Atherosclerosis Research, Department of Medicine, Karolinska Institute, CMM L8:03, 171 76, Stockholm, Sweden; 3 Transgenic Unit, Biomedical Servives Division, Department of Medicine, Lund University, Sölvegatan 19, 223 62, Lund, Sweden; 4 Clincal Chemistry, Department of Laboratory Medicine, Lund University, Klinikgatan 19, 222 42, Lund, Sweden; 5 Current address: Novo Nordisk A/S, Novo Nordisk Park, E9.2.22, 2760, Måløv, Sweden

## Abstract

**Introduction:**

Collagen-induced arthritis (CIA) is a mouse model for rheumatoid arthritis (RA) and is induced after immunization with type II collagen (CII). CIA, like RA, is an autoimmune disease leading to destruction of cartilage and joints, and both the priming and inflammatory phases have been suggested to be dependent on proteases. In particular, the cysteine proteases have been proposed to be detrimental to the arthritic process and even immunomodulatory. A natural inhibitor of cysteine proteases is cystatin C.

**Methods:**

Cystatin C-deficient, sufficient and heterozygous mice were tested for onset, incidence and severity of CIA. The effect of cystatin C-deficiency was further dissected by testing the inflammatory effector phase of CIA; that is, collagen antibody-induced arthritis model and priming phase, that is, T cell response both *in vivo *and *in vitro*. In addition, in order to determine the importance of T cells and antigen-presenting cells (APCs), these cell populations were separated and *in vitro *T cell responses determined in a mixed co-culture system. Finally, flow cytometry was used in order to further characterize cell populations in cystatin C-deficient mice.

**Results:**

Here, we show that mice lacking cystatin C, develop arthritis at a higher incidence and an earlier onset than wild-type controls. Interestingly, when the inflammatory phase of CIA was examined independently from immune priming then cystatin C-deficiency did not enhance the arthritis profile. However, in line with the enhanced CIA, there was an increased T cell and B cell response as delayed-type hypersensitivity reaction and anti-CII antibody titers were elevated in the cystatin C-deficient mice after immunization. In addition, the *ex vivo *naïve APCs from cystatin C-deficient mice had a greater capacity to stimulate T cells. Interestingly, dendritic cells had a more activated phenotype in naïve cystatin C-deficient mice.

**Conclusions:**

The lack of cystatin C enhances CIA and primarily affects *in vivo *priming of the immune system. Although the mechanism of this is still unknown, we show evidence for a more activated APC compartment, which would elevate the autoimmune response towards CII, thus resulting in an enhanced development of chronic arthritis.

## Introduction

Rheumatoid arthritis (RA) is a chronic inflammatory disease causing cartilage and bone destruction in the joints. Interestingly, it is believed that in the inflamed joint the papain-like cysteine proteases, especially cathepsin B, H, L, S and K contribute to the tissue damage [1-5]. Hence, cysteine proteases have been highlighted as potential drug targets to treat tissue degenerative and inflammatory processes [6]. The degradation of the tissue in the joints is clearly mediated by proteolytic activities; however, the specific roles of the different enzymes are still largely unknown. Under physiological conditions the protease activity of these papain-like cysteine proteases are regulated by the cystatins. Cystatin C belongs to the cystatin superfamily 2 and is a potent inhibitor of cathepsins B, H, K, L and S. It is a secreted protein, produced by most nucleated cell types; hence, it is present in all investigated biological fluids. Since cystatin C is a secreted protein, its major site of function is in the extracellular compartment [7,8].

Cystatins, and in particular cystatin C, have been shown to be involved in many biological events and have not always been related to protease inhibition; examples include a neural stem cell factor [9], osteoclast differentiation [10], pathophysiological process in brain ischemia [11] as well as in atherosclerotic plaque development [12,13]. In relation to arthritis, cystatin C has been found to be the most prominent cystatin in synovial fluid of RA patients and that RA patients have significantly lowered levels of cystatin C in circulation [14]. In addition, cystatin C has been shown to enhance fibroblast and smooth muscle cell proliferation and neutrolphil function [15-17]. With this diverse range of possible functions of cystatin C we wished to investigate cystatin C involvement in an *in vivo *autoimmune process in a well-defined animal model.

Collagen induced arthritis (CIA) has been extensively used as an animal model for human RA, and is induced by immunization with type II collagen (CII). Development of CIA has been shown to be B and T cell dependent [18-20]. Furthermore, T cell responses to CII and, consequently, susceptibility to CIA is genetically linked to the MHC class II A^q ^molecule [21]. Interestingly, cathepsin K is one of the few proteases with the capacity to degrade native collagen type I and II [22]. Antigen presentation is an important requirement for the immune response and, indeed, in CIA, the efficiency of presenting certain antigens may enhance the disease profile or may lead to immune tolerance and, thereby, protection against arthritis [23]. Therefore, this delicate balance between disease susceptibility and tolerance can, in part, be regulated by APC. Interestingly, it has been shown that immature dendritic cells (DC) express cystatin C to modulate cysteine protease activity as well as the expression of MHC class II molecules [24]. We have previously shown that the DCs of the epidermis, Langerhans cells (LC), are deficient in presenting CII but not other antigens tested [25]. This phenomenon is an exception from the rule, as DC are highly efficient in presenting antigens and are regarded to be the dominating APC in priming the immune system. Noteworthy, is that this poor presentation of CII could be overcome both *in vitro *and *in vivo *by treating the LC with synthetic cysteine protease inhibitors [26]. These synthetic cysteine protease inhibitors are believed to have a protease inhibitory spectrum similar to that of cystatin C. These studies, however, were not able to directly assess the physiological role of cystatin C. Hence, the aim of the present investigation was to determine the role of cystatin C *in vivo *in terms of arthritis susceptibility and severity.

## Materials and methods

### Mice

The targeted deletion of the cystatin C gene was originally made in an embryonic stem cell line derived from the 129/Sv mouse strain, as described previously [27]. The targeted gene was then backcrossed onto the B10.Q background 10 generations and then intercrossed. In all investigations age and sex matched cystatin C-deficient mice were compared with wild type B10.Q generated in the final intercross. B10.Q mice originated from professor Jan Klein, Tübingen, Germany, and have been maintained within Professor R. Holmdal's unit and this strain is now denoted B10.Q/rhd. To exclude a role for 129 linked genes; F1 mice were generated by crossing cystatin C-deficient mice or wild type B10.Q/rhd with the 129/Sv strain. All mice were housed at Unit for Medical Inflammation Research, Lund University, and experimental procedures were approved by the local (Lund-Malmö region, Sweden) animal ethics organization.

### Antigens

Rat CII was prepared from Swarm chondrosarcoma by limited pepsin digestion, or from lathyritic chondrosarcoma and purified as described earlier [28]. The CII protein was dissolved in 0.1 M acetic acid and stored at 4°C. ConA (Sigma-Aldrich, Steinheim, Germany) was dissolved in PBS and stored at -20°C.

### Induction and evaluation of collagen induced arthritis (CIA)

Mice 8 to 16 weeks of age were immunized at the base of the tail with 100 μg/ml of CII emulsified 1:1 in Complete Freunds Adjuvant (CFA) (Difco, BD, Franklin Lakes, New York USA). A boost injection of CII 50 μg/ml in Incomplete Freunds Adjuvants (IFA, Difco) was given 35 days after immunization. Arthritis was evaluated blindly using a scoring system based on the number of inflamed joints in each paw, inflammation defined by swelling and redness. A maximum score of three points per paw resulted in 0 to 12 points for each mouse as has previously been described in detail [29].

### Sera analyses

For quantification of anti-CII antibodies 96-well plates (Corning Costar, Corning, New York, USA) were coated overnight at 4°C with 10 μg/ml of CII in PBS. Washings were performed using Tris-buffered saline (pH 7.4) containing 0.1% Tween 20 (Tris/Tween). The sera were diluted in wash buffer and tested in duplicate. The amount of bound IgG antibodies was determined after incubation with polyclonal goat anti-mouse IgG mAb conjugated with horseradish peroxidase (Jackson, Immunoresearch Laboratories, West Grove, Pennsylvania, USA). ABTS Tablets (Hoffmann-La Roche, Basel Swissland) were used as chromogenic substrate and absorbance at 405 nm was measured in a Titertec multiscan spectrophotormeter.

### Induction and evaluation of CII antibody induced arthritis (CAIA)

Arthritis was induced in three-to-five-month-old male mice with an anti-CII antibody cocktail containing the two antibodies; M2139 and CIIC1, binding to J1 and C1^1 ^epitopes of CII, respectively. A total dose of 9.0 mg was given each mouse i.v as described earlier [30]. On Day 10, LPS (25 μg/mouse) was injected i.p in all mice in order to enhance the incidence and severity of the disease. Mice were examined daily for arthritis development before and after LPS treatment for a total of 21 days. Arthritis was evaluated blindly based on the number of inflamed joints in each paw, where inflammation was defined by swelling and redness. A maximum score of three points per paw resulted in 0 to 12 points for each mouse as has previously been described in detail [29].

### Delayed type hypersensitivity response (DTH)

Mice were immunized with 100 μg/ml of CII emulsified in CFA at the base of the tail. On Day 10 after immunization, the mice were challenged with CII (dissolved in 0.1 M acetic acid) mixed with PBS and injected i.d. into the right ear (30 μg in 0.02 M acetic acid per mouse). The left ear was injected with the vehicle (PBS/0.02 M acetic acid) alone and used as a control. Ear thickness was measured 24, 48 and 72 hours after challenge and the difference of ear thickness between right and left ear was calculated.

### T lymphocyte activation assay

T lymphocyte activation assay was performed using lymphocytes or splenocytes either as *in vitro *re-call assay using immunized mice or as *in vitro *T cell priming assay using naïve cells. In both cases B10.Q/rhd wild type and cystatin C-deficient mice aged 8 to 16 weeks were used. Single cell suspension of either naïve mice or mice immunized 10 days prior (immunized as above in CIA induction) were cultivated in Dulbecco's modified Eagle medium (DMEM, Gibco, Invitrogen Carlsbad, California, USA) containing 10% FCS, 10 μM β-mercaptoethanol, 10 mM HEPES, penicillin, and streptomycin (complete media, cDMEM) in humidified incubator at 37°C in 7.5% CO_2_. Cells were stimulated with 25 μg/ml of lathyritic type II collagen (only cells from immunized mice) or with 3 μg/ml of ConA for 72 hours. An aliquot of supernatant was taken after 24 h of stimulation in order to detect IL-2 production. IL-2 and IFN-γ responses were measured by ELISA, with anti-IFN-γ, (5 μg/ml, clone An18 prepared in-house) anti-IL-2, (2 μg/ml, clone JES-6-1A12 prepared in-house) as capturing antibodies and biotinylated anti-IFN-γ, (0.6 μg/ml, clone R46-A2, MABTECH, Nacka Strand, Sweden), and biotinylated anti IL-2 (1 μg/ml, clone JES6-5H4, MABTECH) as detection antibody. 96-well plates (Corning Costar) were coated overnight at 4°C with capturing antibodies, plates were then incubated with 2% BSA in PBS for one hour to block unspecific binding. Samples were added and incubated at room temperature for two hours or at 4°C overnight. Finally, the plates were incubated for two hours at room temperature with detecting antibody in PBS containing 10% FCS and 0.1% Tween. For detection the plates were incubated with europium-avidin followed by enhancement buffer according to the manufacturers' instructions and fluorescent intensity measured using a fluorometer (Wallac Oy EG & G, PerkinElmer, Waltham, Masschusetts, USA). Proliferation was measured via Thymidine incorporation where the cell culture was pulsed with (^3^H) Thymidine for final 15 to 18 hours of cultivation. The cells were harvested in a Micromate 196 cell harvester (Canberra Packard, Schwadorf, Austria) and the radioactivity determined in a Matrix™ direct β-counter (Canberra Packard).

### Cell purification prior to T lymphocyte activation assay

In this system, splenic T cells from cystatin C-deficient mice were co-cultured with splenic antigen-presenting cells (APCs) either form cystatin C-deficient or wild type mice, and wild type splenic T cells were co-cultured with splenic APCs either from cystatin C-deficient or wild type mice. T cells were separated from the APC population by using the mouse CD4- and CD8 Dynabeads^® ^FlowComp™ system (Invitrogen™ Carlsbad, California, USA), according to the manufacturer's instructions. The T cell population was 95% pure (as observed by FACS, data not shown). Selected T cells from each mouse were used individually. In order to obtain the APC population, those cells that were CD4/CD8 negative were subjected to an additional purification step where they were first incubated with anti-CD4 biotinylated antibody (GK1.5, prepared in-house), followed by Dynabeads^® ^Biotin Binder, Invitrogen™, in order to deplete the remaining T cells. This population was then pooled and used as APCs in co-culture with purified T cells. In all experiments 2.5 × 10^5 ^T cells/well were co-cultured with 4 × 10^5 ^APCs in the same well. The level of IL-2 production was measured after 24 h and the IFN-γ concentration was measured after 72 h of stimulation with 3 μg/ml of ConA as described above.

### Flow cytometry

Single cell suspension of spleens from naïve cystatin C-deficient and wild type controls were prepared and red blood cells were lysed with 0.84% NH_4_Cl_2_. Cells were seeded (1 × 10^6 ^per well) into 96-well plates and stimulated with either cDMEM alone or with 2 ug/mL of ConA (Sigma-Aldrich) in cDMEM in a humidified incubator at 37°C in 7.5% CO_2 _and cultivated for 24 hours at 37°C. After 24 hours incubation or freshly prepared splenocytes from cystatin C-deficient and wild type controls cells were washed and Fc receptors blocked, using 24.G2 (prepared in-house), cells were stained for cell specific and activation markers. T cell staining consisted of: FITS-CD3 (clone 145-2C11), Pe-Cy5-CD4 (clone GK1.5), PerCP-Cy5.5- CD8a (clone 53-6.7), APC-Cy7-CD25 (clone PC61), PE-FR4 (clone TH6), PE-Cy7-CD69 (clone H1.2F3), and live cells were determined using ViDye Violet. APC staining consisted of: Alexa Fluor^® ^700-CD45R/B220 and CD19 (clones RA3-6B2 and 6D5 respectively), Pacific blue-CD11b (clone M1/70), Pe-Cy7-CD11c (clone N418), Pe-Cy5-F4/80 (clone BM8), APC-Cy7-Ly-6G/Ly-6C (GR-1, clone RB6-8C5), Alexa Fluor^®^488-MHC II (clone M5/114 purchased from BD, Franklin Lakes, USA), PE-CD40 (stained intracellular using BD intracellular staining kit according to the manufacturers recommendations, clone IC10) or PE-CD80 (clone 16-10A1) Alexa Fluor^® ^647-CD54 (ICAM, Clone YN1/1.7.4) or Alexa Fluor^® ^700-CD86 (clone GL-1). For both dilution of antibodies and cells suspension, PBS with 0.1% BSA (Sigma-Aldrich) and 0.01% sodium Azide (Sigma-Aldrich) was used. However, for intracellular staining against CD40 the BD intracellular staining kit was used. All antibodies were purchased from BioLegend (San Diego, California USA) unless indicated otherwise and ViDye was purchased from Invitrogen. All antibodies and Vidye were titrated using the according to the manufacturers recommendations. The cells were then analyzed by flow cytometry (LSRII; BD).

### Antigen presentation assays

The antigen-presenting capacity of isolated APC was determined by their ability to stimulate MHC A^q^-restricted T-cell hybridoma, namely HCQ.10 clone that responds to CII and the galactose-peptide 256-270 [31]. Langerhans cells were prepared from mouse ears as described earlier [32]. Bone marrow derived dendritic cells (BMDCs) were prepared as described [33]. B cells were separated from lymph nodes from mice immunized 10 days prior, using anti-mouse CD45R/B220 (clone RA3-6B2) conjugated to microbeads, as described by the manufacturer (Miltenyi Biotec, Bergisch Gladbach, Germany). Peritoneal exudate cells were collected by peritoneal lavage where macrophages were enriched by depleting B cells using anti-mouse CD45R/B220 (clone RA3-6B2) microbeads (Miltenyi Biotec). Spleen DC were isolated by dissecting the spleen into small pieces, with 5 ml DMEM containing antibiotics, 2% FCS, 0.5 mg/ml of collagenase type IV (Worthington Biochemical Corp. Lakewood USA) and 50 U/ml of DNase (Deoxyribonuclease 1 from bovine pancreas, Sigma-Aldrich), and incubated at 37°C for 40 to 60 minutes with agitation. Digested material was passed through a 70 μm cell strainer (Falcon, BD Franklin Lakes USA), remaining tissue pieces were minced through the strainer using a syringe plunger. Cells were washed in PBS containing 2% FCS and 2 mM EDTA. Non-specific binding was blocked with anti-FcR mAb (clone 2.4.G2 prepared in-house) for 15 minutes on ice, and DC were enriched using positive selection with anti-CD11c microbeads (Miltenyi Biotech) and twice passed through LS magnetic column (Miltenyi Biotech). Antigen presenting assay was performed by co-cultivating T-cell hybridoma cells (50 × 10^3^) with the syngeneic APC and antigen in a total volume of 200 μl in flat-bottomed 96-well plates (Nunc, Thermo Scientific, Rochester, New York, USA). After 24 h culture, 100 μl aliquots of the supernatants were cultured with 1 × 10^5 ^cells/well of an IL-2 dependant murine cytotoxic T cell line (CTLL), in a total volume of 200 μl for 24 h and the CTLL cells were then pulsed with (^3^H) Thymidine for an additional 15 to 18 h. The cells were harvested in a Micromate 196-cell harvester (Canberra Packard, Meriden, Connecticut, USA) and the radioactivity determined in a Matrix™ direct β-counter (Canberra Packard). In addition, a sandwich ELISA was used to determine IL-2 concentration as described above in section "T lymphocyte activation assay assay".

### Statistical analysis

The Prism GraphPad software program (La Jolla, California, USA) was used for the statistical analysis. All mice were included for calculation for arthritis susceptibility, whereas severity was determined with affected mice only. The Mann-Whitney U test or Students *t*-test was applied to evaluate statistical differences or for disease incidence Fisher's exact test was used.

## Results

### Cystatin C-deficient mice have an increased incidence and earlier onset of arthritis

**Figure 1:**
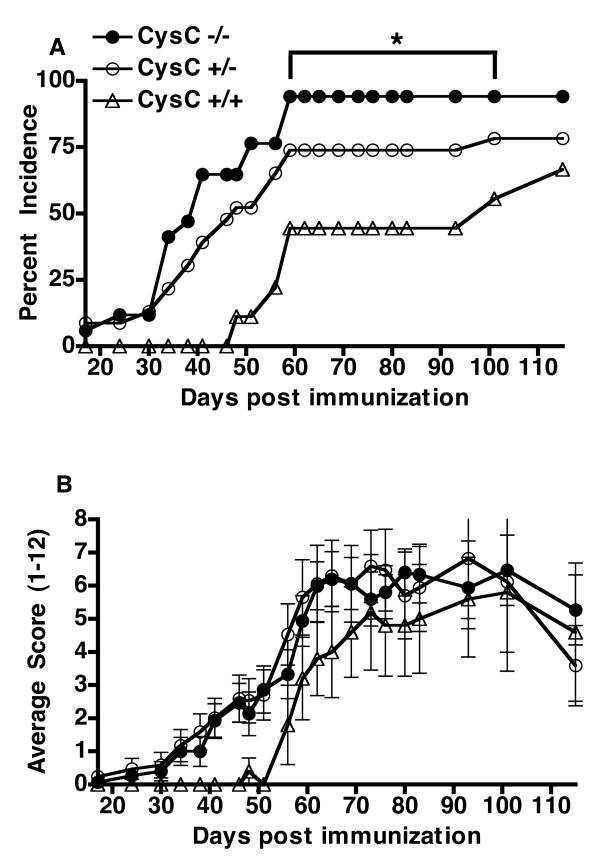
**Cystatin C-deficient mice have a higher incidence of arthritis but have a similar arthritic score**. **A**, Cystatin C-deficient mice (Cyst C-/-, *n *= 17) had a greater cumulated arthritis incidence (that is, all mice that have shown signs of arthritis at any time point are included as diseased individuals) compared to heterozygous (Cyst C-/+, *n *= 23) and wild type litters Cyst C+/+, *n *= 9). Data shown are from one representative experiment out of two separate experiments (combined data shown in Table 1). * *P-*value < 0.05 as tested in Fisher's exact test. **B**, Cystatin C-deficient mice (*n *= 15) had a similar arthritic severity compared to heterozygous (*n *= 17) and wild type littermate controls (*n *= 5) when affected mice were compared (mice were excluded if no clinical signs of arthritis was seen after 115 days post immunization).

To investigate the role of cystatin C in the development of arthritis and, hence, its role in the immune response towards CII, cystatin C-deficient, cystatin C heterozygous- and cystatin C-sufficient B10.Q/rhd wild type littermates were immunized with CII in CFA. The cystatin C-deficient mice were more prone to develop arthritis with a significantly higher incidence compared to the wild type controls (Figure 1A, Table 1) arguing for a protective role of cystatin C. Furthermore, cystatin C-deficient mice developed arthritis earlier than the wild type control mice, but there was no significant difference in the severity of arthritis as the maximal score was similar among all groups (Figure 1B, Table 1). Surprisingly, no difference concerning disease activity, nor incidence, could be observed between homozygous and heterozygous cystatin C-deficient mice. This could indicate a dose level effect of cystatin C.

**Table 1:** CIA susceptibility in Cystatin C-deficient mice compared to heterozygous and wild type littermates

Genotype	**Arthritis incidence**^**#**^**, number with/number without (%)**	Maximum score, mean ± SEM	**Day of onset**^**†**^**, mean ± SEM**	**Disease Duration**^**† **^**mean ± SEM**	**Recovery**^**§ **^**rate: number recovered/number active arthritis (%)**	Area under curve
Cystatin C -/-	25/8 (75*)	6.917 ± 0.72	44.57** ± 2.88	67.96* ± 5.29	4/21 (16*)	259.9
Cystatin C +/-	23/12 (65)	7.955 ± 0.83	47.91** ± 4.23	54.41 ± 5.90	9/14 (39)	230.8
Cystatin C +/+	12/13 (48)	6.500 ± 1.15	69.67 ± 7.78	38.08 ± 8.06	6/6 (50)	156.4

# Cumulative incidence, that is, all mice that have presented with arthritis. Chi-square test was used for determining statistical significance; † Mann Whitney test was used for determining statistical significance; § Percent recovery was calculated by: (number of mice that had presented arthritis but did not have active arthritis at Day 115 divided by the total number of mice that had presented arthritis during the 115-day period) multiplied by 100. Chi-square test was used for determining statistical significance; * *P-*value less than 0.05; ** *P-*value less than 0.01

**Figure 2:**
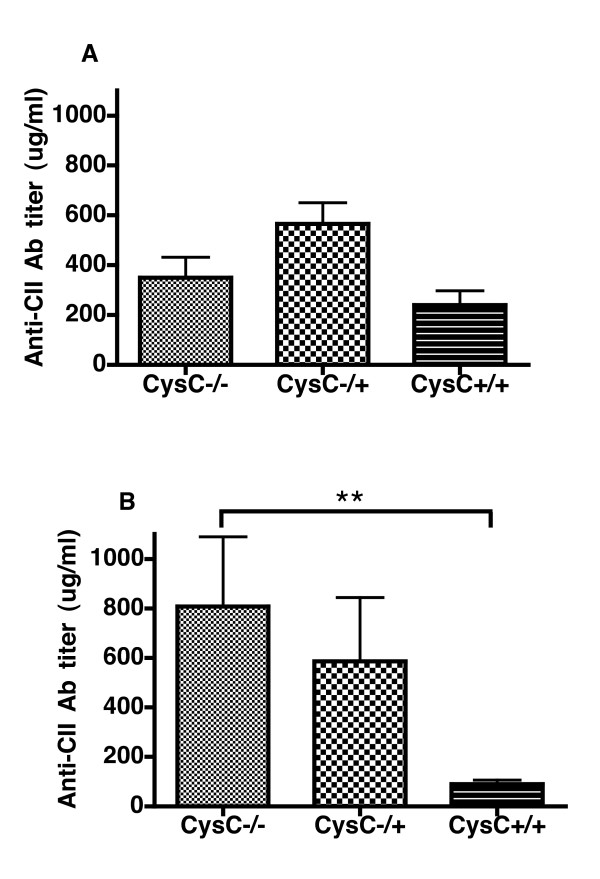
**The anti-CII IgG antibody titers are elevated in the cystatin C-deficient mice**. Mice were bled at Day 34 (**A**) before boost immunization with CII/IFA, and at the end of the experiment at Day 115 (**B**). There was a statistically significant difference between the antibody production of cystatin C-deficient mice (Cyst C -/-) and the wild type controls (Cyst C +/+) at Day 115. ** *P-*value < 0.01 as tested with Mann-Whitney.

The CII-reactive antibody response, which is known to correlate with disease onset and severity, was measured at both day 35 and at day 115. Surprisingly, at day 34 there was a trend but no significant difference in anti-CII antibody titers between cystatin C-deficient and wild type controls in terms of anti-CII antibody titers (Figure 2A). However, at day 115, which reflects the chronic phase of the disease, there was a significant difference between cystatin C-deficient and wild type controls. In fact, in the wild type mice, antibody titers at day 115 had dropped dramatically compared to day 34, indicating that the disease is in the process of resolving. However, this was not the case in the cystatin C-deficient mice as antibody levels were higher at day 115 than at day 34 (Figure 2B), indicating a sustained immune response. Further, a chronic disease profile was observed in the cystatin C-deficient mice compared to wild type controls as there was a significantly longer disease duration and fewer mice recovered from arthritis (Table 1). Hence, cystatin C clearly had a protective role in CIA, as deficient mice were more prone to a chronic arthritis and had a sustained autoreactive antibody response.

### Effector phase of arthritis is similar between cystatin C-deficient and control mice

**Figure 3:**
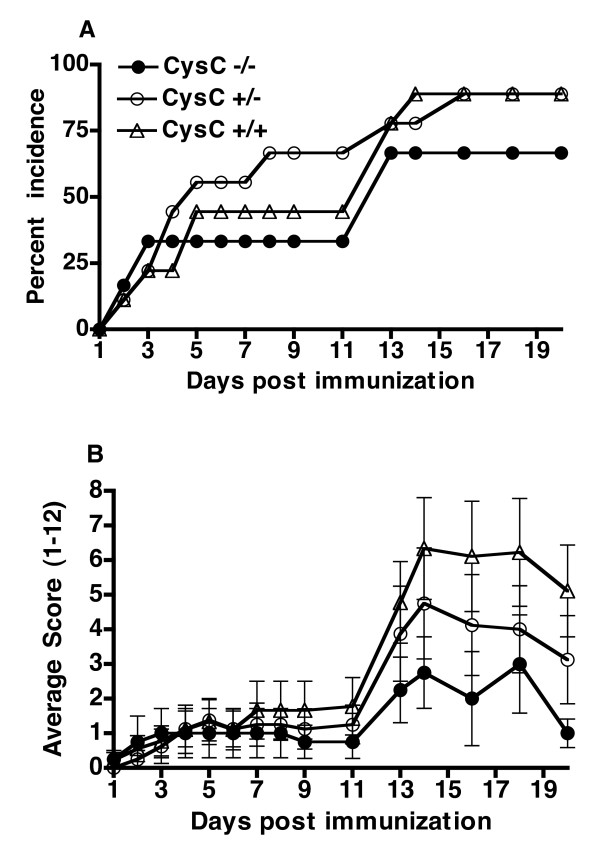
**Effector phase is similar between cystatin C-deficient and control mice**. CAIA was induced with two monoclonal anti-CII antibodies by i.v injection into cystatin C-deficient (Cyst C -/-, *n *= 6), cystatin C heterozygous (Cyst C -/+, *n *= 9) and WT littermate controls (Cyst C +/+, *n *= 10). **A**, No significant difference was seen in incidence of arthritis. **B**, There was also no difference in arthritis severity when comparing affected mice (Cyst C -/- *n *= 4, Cyst C -/+ *n *= 8, Cyst C +/+ *n *= 9, mice were excluded if no clinical signs of arthritis was seen after 20 days post antibody injection). The error bars indicate SEM.

Since the cystatin C-deficient mice displayed a more sustained antibody synthesis compared to wild type littermates (high antibody level at Day 115), the question then arises whether cystatin C has an effect on priming of the immune response or is more involved in the inflammatory effector phase of the disease. The effector phase of CIA can be observed separately from immune priming by administering arthritogenic anti-CII antibodies, that is, CAIA. Conversely to the effect of cystatin C-deficiency in CIA, incidence of arthritis and day of arthritis onset were not enhanced in cystatin C-deficient mice in the CAIA model when compared to wild type mice (Figure 3). This argues for cystatin C influencing the autoimmune priming rather than the inflammatory effector phase in CIA.

### Enhanced delayed type 1 hypersensitivity (DTH) reaction response in cystatin C-deficient mice

**Figure 4:**
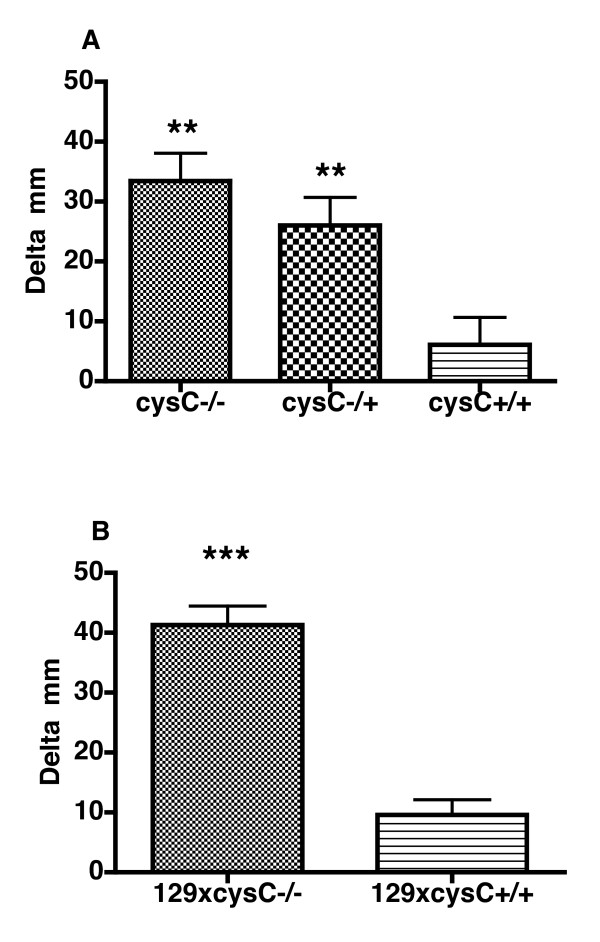
**Cystatin C-deficient mice show a stronger DTH reaction**. **A**, Cystatin C-deficient mice (Cyst C -/-, *n *= 11) developed a significantly stronger DTH reaction towards CII compared to the wild type controls (Cyst C +/+, *n *= 11) 48 h after challenge, and so did the heterozygous cystatin C-deficient mice (Cyst C -/+, *n *= 11). **B**, The observed enhanced DTH was due to cystatin C-deficiency and not 129/Sv linked genes as F1 intercross between 129/Sv x cystatin C-deficient mice (*n *= 12) had a significantly enhanced DTH compared to the F1 intercross between 129/Sv x B10.Q/rhd (*n *= 12) 48 h after challenge. Significance was calculated with Mann-Whitney test where ** *P-*value < 0.01 and *** *P-*value < 0.001.

Since cystatin C-deficiency did not alter the inflammatory effector phase and the fact that both cystatin C-deficient and heterozygous mice developed arthritis earlier than the wild type controls, there was an indication that cystatin C-deficiency had an effect on priming the immune response. We, therefore, wished to investigate whether the T cell response to CII was altered in cystatin C-deficient mice. The delayed-type hypersensitivity (DTH) reaction is a T cell recall response to the immunized antigen. Hence, mice were immunized with CII and challenged in the ear with CII or PBS 10 days later. Ear thickness was measured 48 and 72 hours after the challenge. After 48 hours the cystatin C-deficient mice already had an enhanced response (Figure 4A). These results are in line with the initial findings of an enhanced CIA disease profile of the cystatin C-deficient mice.

### The observed DTH effect is dependent on cystatin C and not 129/Sv-linked genes

Since the cystatin C-deficient mice were originally produced in the 129/Sv strain we could not, despite extensive backcrossing, exclude that 129/Sv genes in the linked fragment containing the null-mutation had an influence on our results. It was particularly important to clarify the possible influence of such genes, since the enhancing effects on CIA and anti-CII B and T cell responses *in vivo *were also seen in heterozygous cystatin C-deficient mice. Therefore, a DTH response was tested in an F1 intercross between 129/Sv and B10.Q/rhd wild type mice (controls) and compared to an F1 intercross between 129/Sv and the B10.Q/rhd backcrossed cystatin C-deficient mice (experimental group). Reassuringly, the experimental group showed a significantly stronger DTH-reaction than the corresponding controls (Figure 4B). This shows that the cystatin C-deficiency, and not a dominant 129/Sv-derived gene effect, caused the observed enhanced DTH response.

### Cystatin C-deficient APCs compared to wild type have a greater propensity to stimulate cystatin C sufficient T cells in T lymphocyte activation assay

**Figure 5:**
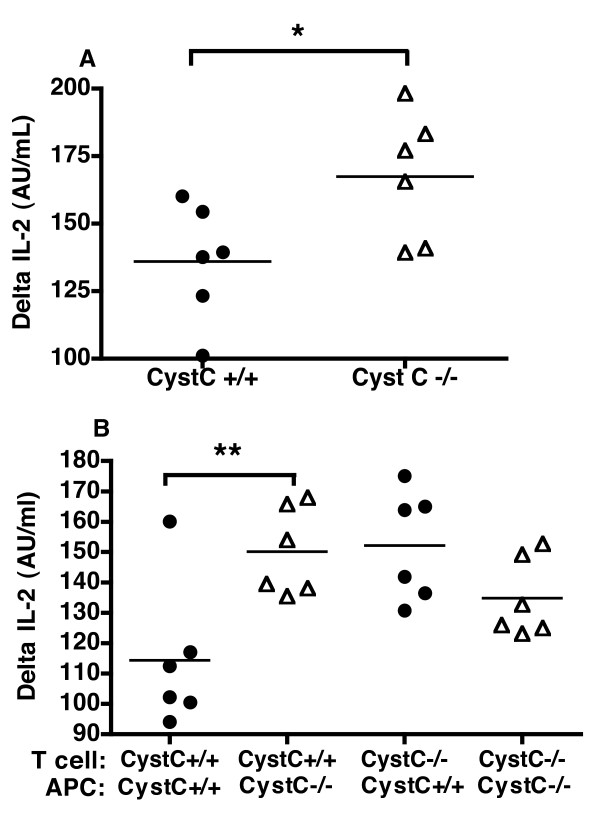
**Cystatin C-deficient APCs have a greater propensity to stimulate cystatin C-sufficient T cells**. Splenocytes were prepared from either cystatin C-deficient mice or wild type mice and stimulated *in vitro *with either media alone or with 2 ug/mL ConA (change in production level (delta) is shown, where the response to media alone is deducted from the ConA response). Cystatin C-deficient splenocytes mice (CysC-/-) had a significantly higher production of IL-2 (**A**) after 24 h Cystatin C-deficient APCs were able to enhance the IL-2 production of T cells from wild type mice, where as there was no difference in IL-2 production in cystatin C-deficient T cells when co-cultured with either wild type (CysC+/+) or cystatin C-deficient APCs (**B**). However, when wild type APCs where co-cultured with T cells then the cystatin C-deficient T cells produced more IL-2 than wild type T cells. Six mice were used per group, statistical significance was calculated by Students *t*-test where * *P-*value < 0.05 and ** *P-*value < 0.01.

With the finding that mice deficient in cystatin C had an enhanced DTH, we wished to observe whether this effect was due to T cells or APCs or a combination of both. In order to investigate this we used an *in vitro *T lymphocyte activation assay. Surprisingly, we observed that the recall response of immunized cystatin C-deficient lymphocytes or splenocytes did not differ significantly from wild type cells in terms of T cell proliferation, IL-2 or IFN-γ production (data not shown). Therefore, we considered the possibility that there was a difference in the initial T cell priming and not in the recall response *in vitro*. Indeed, the splenocytes from naïve cystatin C-deficient mice showed an enhanced production in IL-2 after 24 h culture (Figure 5) but no difference in T cell proliferation or IFN-γ production after 72 h (data not shown) when stimulated with a polyclonal stimulant ConA. Furthermore, if purified wild type T cells were co-cultured with purified APC from cystatin C-deficient mice then IL-2 production was enhanced compared to purified wild type T cells co-cultured with wild type APC, but again no difference in IFN-γ production was observed (Figure 5). Interestingly, the T cells from cystatin C-deficient mice displayed an enhanced IL-2 production at a significantly higher level than cystatin C-sufficient T cells irrespective of the genotype of the APC's used. This indicates that the T cells in cystatin C-deficient mice have a constitutively lowered activation threshold or have been conditioned *in vivo*, but the mechanism of this is beyond this study.

### DC's from naïve cystatin C-deficient mice are more activated

In view of the fact that the APCs from mice deficient for cystatin C had an enhanced ability to stimulate T cells *in vitro*, we were interested to see if there was a difference in expression of MHC class II or activation markers between cystatin C-deficient and wild type mice. The activation markers investigated included CD80, CD86, CD40 and ICAM, which are co-stimulatory molecules known to aid in T cell activation. Splenocytes of naïve mice as well as splenocytes stimulated with ConA and media alone for 24 h were analyzed by flow cytometry. In freshly isolated splenocytes from naïve mice there was no difference observed in cell composition with the similar percentage of B cells (CD19 and CD45RB+), DC (CD11c^+^, F4/80 antigen^- ^and LyCG^-^), macrophages (F4/80 antigen^+^, CD11c^- ^and Ly6G^-^), neutrophils (CD11b^++ ^Ly6G^+^), CD4^+ ^(CD3^+^CD4^+^) and CD8^+ ^(CD3^+^CD8^+^) T cells, and regulatory T cells (CD4^+^CD25^+^FR4^+^) between cystatin C-deficient and wild type mice (data not shown).

**Figure 6:**
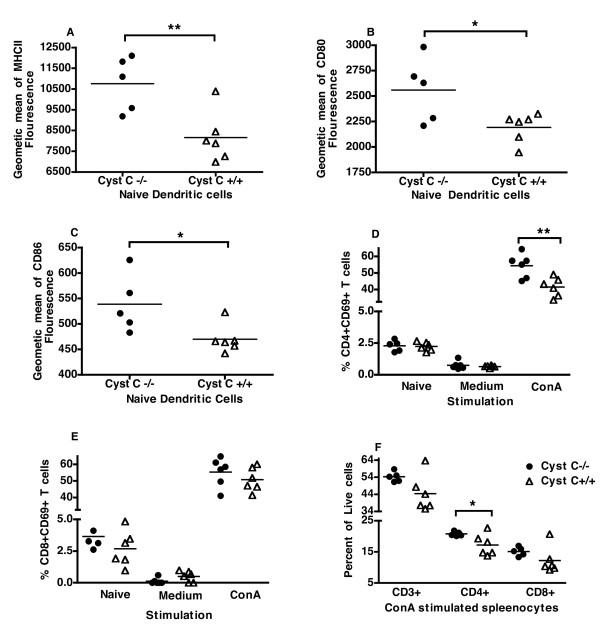
**Cystatin C-deficient DCs have increased expression of MHCII and Co-stimulatory molecules, and more activated T cells**. Splenocytes from naïve mice or splenocytes stimulated with ConA or media were analyzed by flow cytometry. Naive DCs from cystatin C-deficient (CysC-/-) mice expressed higher intensity of MHCII (**A**) CD80 (**B**) and CD86 (**C**). A greater percent of CD4^+ ^T cells expressed CD69 in cystatin C-deficient mice than wild type mice (CysC+/+) when stimulated with ConA for 24 h (**D**) but not CD8+ T cells. (**E**), In line with this there was a higher percent of CD4^+ ^T cells in cystatin C-deficient mice and a tendency for a higher percent of CD8^+ ^and total T cells (**F**). Six mice were used per group, statistical significance was calculated by Students *t*-test where * *P-*value < 0.05 and ** *P-*value < 0.01.

Splenic DCs from naïve cystatin C-deficient mice, but not any other of the investigated cell populations, had an increased expression of MHCII, CD80 and CD86 (Figure 6A-C) but not ICAM or CD40 (data not shown) compared to naïve wild type mice. Interestingly, upon ConA stimulation, there was no statistical difference in MHCII or CD80 expression and CD86 was in fact expressed higher on wild type cells (data not shown). However, the kinetics of this expression was not determined.

The activation of T cells from the cystatin C-deficient and wild type mice was also investigated and as we would have predicted from the DTH results, and *in vitro *T lymphocyte activation experiments, the cystatin C-deficient CD4^+ ^T cells were more activated (higher percentage of CD4^+ ^T cells expressed CD69, Figure 6D). However, there was no significant increase in CD69 expressing CD8^+ ^cells (Figure 6E). In line with the earlier results there was an increase in percent of CD4^+ ^T cells (Figure 6F) after stimulation with ConA. There was also a tendency toward increase in T cells (CD3^+ ^cells *P *= 0.08) as well as in CD8+ T cells (*P *= 0.18), but the absolute number of cells was not calculated. Similar to the freshly prepared splenocytes from cystatin C-deficient and wild type mice there was no difference in cell composition with the similar percentage of B cells, DC, macrophages, neutrophils and regulatory T cells in ConA stimulated cells (data not shown).

### Antigen presentation is not enhanced in APCs from cystatin C-deficient mice

Cystatin C is an inhibitor of the cathepsins proteases that have a crucial role in the cleaving of proteins for antigen presentation; therefore, it is conceivable that cystatin C would also have a prominent role in antigen presentation. Furthermore, since naïve cystatin C-deficient DC expressed a higher degree of MHC II molecules, it was plausible that antigen presentation was enhanced in cystatin C-deficient mice. Therefore, in order to remain as close to the DTH setting, CII (without adjuvant) was injected i.d in the ear of cystatin C-deficient and wild type mice. Six hours later epidermal antigen presenting cells were prepared, and tested for APC activity to CII specific T cell hybridomas (HCQ.10). However, there was no increased hybridoma response between the DCs derived from cystatin C-deficient or wild type mice. This was also the case when naïve skin epidermal APC's, spleen DC, isolated macrophages, B cells and bone morrow derived DC were stimulated with CII *in vitro *(data not shown). Therefore, the enhanced stimulatory capacity of cystatin C-deficient APC is not due to a direct increase in antigen processing.

## Discussion

The natural inhibitor of cysteine proteases, cystatin C, has been proposed to have a protective role in chronic inflammatory disorders such as RA and atherosclerosis. In the current study we demonstrate that cystatin C plays an important role in the development of an autoimmune B and T cell response to type II collagen, which in turn leads to the early development of a chronic CIA, rather than having an effect on the inflammatory effector phase. Our findings clearly show that cystatin C has a direct autoimmune-regulating function and to our knowledge, this has not been previously demonstrated in an *in vivo *model.

With the use of the CAIA model, we were able to pinpoint whether cystatin C had a principal role in the priming or effector inflammatory phase. The CAIA model involves the administration of CII-specific antibodies that bind to the joint cartilage resulting in an inflammatory response that is driven by activated macrophages and neutrophils [34]. The fact that CAIA is driven by neutrophils was of particular interest as cystatin C has been previously shown to effect neutrophil function [15,16]. Interestingly, the CAIA was not enhanced in cystatin C-deficient mice, which strongly indicated that the immune priming phase of CIA was altered in cystatin C-deficient mice and that neutrophils were not dramatically affected by cystatin C-deficiency.

In RA, cystatin C has been proposed to have an indirect immuno-modulatory role via the removal of acute phase protein serum amyloid A (SAA) [14]. In the current study we cannot rule out that the lack of cystatin C resulted in higher levels acute phase proteins and, in particular SSA, as this was not measured. Notably, in the CAIA model, LPS is administered to boost severity and incidence of arthritis and it is well known that LPS is one of the most potent enhancers of SSA. Still, there was no enhanced disease profile between cystatin C-deficient and WT mice. Therefore, it is most unlikely that an excessive level of SSA was a prominent mechanism in the exacerbated CIA disease profile of cystatin C-deficient mice.

Since cystatin C-deficient mice did not have an increased inflammatory effector phase, but had and earlier onset with a higher incidence of arthritis, we wished to investigate the priming phase of CIA. Hence, we used a DTH reaction and cystatin C-deficient mice had a robust increase in the recall response. Surprisingly, we were unable to see any difference between cystatin C-deficient and sufficient mice in T cell activation using an *in vitro *T lymphocyte activation assay of immunized mice. However, if naïve splenocytes were stimulated *in vitro *with the polyclonal T cell activator ConA, then there was a clear difference in IL-2 production, but *in vitro *this difference was not reflected in T cell proliferation or IFN-γ production. The fact that there seems to be a contradiction between the *in vivo *and *in vitro *findings is not uncommon and could be due to a vast range of factors. ConA was used in these studies, as naïve T cells are unresponsive to antigens such as CII without prior immunization. However, ConA requires the presence and activation of APCs in order to stimulate naïve T cells, and in this way mimics one aspect of the initial priming of T cells *in vivo*. This use of ConA is not ideal but gives a starting point to start to unravel the complex mechanisms of cytstatin C. In addition to the initial findings of altered IL-2 production, when we separated the APC from the T cell compartment observed that the APC from cystatin C-deficient mice had a greater propensity to stimulate T cells from wild type mice, but that this was only in the first 24 h of T cell activation. This implies that the APC compartment in cystatin C-deficient mice is more activated. Interestingly, the T cells from cystatin C-deficient mice responded more than the wild type T cells, regardless of APC genotype. These observations indicate that the APC from cystatin C-deficient mice have a greater propensity to stimulate T cells, but the cystatin C-deficiency also affects the T cell activation level. These findings need to be further investigated, and an obvious continuation would be the use of CII specific TCR-transgenic mice that are deficient in cystatin C, where the naïve T cell response to CII can be directly investigated *in vitro*. Interestingly, upon flow cytometry fine phenotyping of the cystatin C-deficient mice, we observed that the DC compartment was of a more activated phenotype than the wild type controls. This finding further supports the notion that the APC compartment of the cystatin C-deficient mice is more activated.

An inherent problem when using knock-out mice is the contamination of the genome with the genes derived from the embryonic stem cell (ES) line used to create the mouse. In the case of the cystatin C-deficient mouse the ES line used was derived from the 129/Sv strain. There is, then, even after 10 generations of backcrossing, a risk that the observed DTH and CIA effect is due to 129/Sv genes linked to the targeted locus, and not due to the cystatin C-deficiency. Therefore, we created an F1 cross between 129/Sv and B10.Q/rhd cystatin C-deficient mice, and also an F1 generation between 129/Sv and B10.Q/rhd mice. The DTH of the two F1 groups showed an enhanced DTH reaction in the mice heterozygous for cystatin C-deficiency and this was similar to the DTH reaction of the pure B10.Q/rhd mice carrying one allele of cystatin C-deficiency. Therefore, we could be certain that the observed enhanced DTH response was due to cystatin C-deficiency and not the surrounding 129/Sv genes.

There was very little difference in the cellular component of the cystatin C-deficient and wild type mice where the percentage of T cells and APCs was very similar. Upon further flow cytometry investigations, it could be seen that the DC population in naïve mice were more activated with a higher expression of MHCII and CD80 and CD86. In addition, when spleen cells were stimulated with ConA for 24 h, T cells derived for cystatin C-deficient mice were found to be more activated with a higher expression of CD69. Interestingly, although DCs from cystatin C-deficient mice expressed MHCII to a greater extent than wild type mice, there was no distinct difference in antigen presentation capacity.

## Conclusions

Cystatin C is an abundant protein that is expressed in all nucleated cells, and has been found to be involved in a range of biological functions; therefore, pinpointing the fine mechanism of cystatin C in complex diseases is difficult. However, this study demonstrates that cystatin C has a protective role in CIA and that cystatin C-deficient mice have a chronic disease profile. We postulate here that one plausible mechanism of cystatin C-deficiency could be the enhancement of T cell priming via a more activated APC compartment. The enhanced T cell priming would lead to T cells supplying B cell help that in turn would lead to a greater production of autologous antibodies and a chronic arthritis profile would develop.

## Abbreviations

APCs: antigen-presenting cells; BM: bone marrow cells; BMDC: bone marrow derived dendritic cells; CAIA: collagen antibody induced arthritis; CII: collagen type II; CIA: collagen induced arthritis; CTLL: murine T cell line; DC: dendritic cells; DTH: Delayed-Type Hypersensitivity response; LC: Langerhans cells; RA: rheumatoid arthritis; SAA: serum amyloid A.

## Competing interests

The authors declare that they have no competing interests.

## Authors' contributions

AB and MH contributed equally, were involved in performing the majority of experiments and drafted the manuscript. RM was involved in assisting and planning the CIA experiments and helped to draft the manuscript, KSN was involved in initiating the CAIA experiments and producing the anti-CII antibodies and helped to draft the manuscript. VL and KH designed the screening method for the mice and screened all mice and helped to draft the manuscript. The study was originally designed by RH in collaboration with AG, and both RH and AG helped to draft the manuscript. All authors read and approved the final manuscript.
